# Efficacy and clinical effectiveness of influenza vaccines in HIV-infected individuals: a meta-analysis

**DOI:** 10.1186/1471-2334-6-138

**Published:** 2006-09-11

**Authors:** Julius Atashili, Linda Kalilani, Adaora A Adimora

**Affiliations:** 1 Department of Epidemiology, University of North Carolina, Chapel Hill, NC 27599–7435, USA; 2 Center for the Study and Control of Communicable Diseases (CSCCD), Faculty of Medicine and Biomedical Sciences, University of Yaoundé I, BP 8445 Yaoundé, Cameroon; 3 School of Medicine, University of North Carolina, Chapel Hill, NC 27599–7435, USA

## Abstract

**Background:**

Though influenza vaccines are the cornerstone of medical interventions aimed at protecting individuals against epidemic influenza, their effectiveness in HIV infected individuals is not certain. With the recent detection of influenza strains in countries with high HIV prevalence rates, we aimed at evaluating the current evidence on the efficacy and clinical effectiveness of influenza vaccines in HIV-infected individuals.

**Methods:**

We used electronic databases to identify studies assessing efficacy or effectiveness of influenza vaccines in HIV patients. We included studies that compared the incidence of culture- or serologically-confirmed influenza or clinical influenza-like illness in vaccinated to unvaccinated HIV infected individuals. Characteristics of study participants were independently abstracted and the risk difference (RD), the number needed to vaccinate to prevent one case of influenza (NNV) and the vaccine effectiveness (VE) computed.

**Results:**

We identified six studies that assessed the incidence of influenza in vaccinated HIV-infected subjects. Four of these studies compared the incidence in vaccinated versus unvaccinated subjects. These involved a total of 646 HIV-infected subjects. In all the 4 studies, the incidence of influenza was lower in the vaccinated compared to unvaccinated subjects with RD ranging from -0.48 (95% CI: -0.63, -0.34) to -0.15 (95% CI: -0.25, 0.05); between 3 and 7 people would need to be vaccinated to prevent one case of influenza. Vaccine effectiveness ranged from 27% to 78%. A random effects model was used to obtain a summary RD of -0.27 (95%CI: -0.42, -0.11). There was no evidence of publication bias.

**Conclusion:**

Current evidence, though limited, suggests that influenza vaccines are moderately effective in reducing the incidence of influenza in HIV-infected individuals. With the threat of a global influenza pandemic, there is an urgent need to evaluate the effectiveness of influenza vaccines in trials with a larger number of representative HIV-infected persons.

## Background

Influenza viruses are common causes of acute respiratory illnesses [1]. The recent spread of the H5N1 type in Asia has alerted the public health community to the threat of an influenza pandemic similar to those the world experienced in 1918, 1957 and 1968 [2]. With an estimated 40.3 million people living with HIV infection by the end of 2005[3], it is imperative to evaluate the potential impact of the HIV epidemic on an influenza pandemic and influenza control measures.

Influenza vaccines are the cornerstone of medical interventions aimed at protecting individuals against epidemic influenza. The efficacy of influenza vaccines in HIV infected individuals remains poorly defined. Theoretical safety concerns stem primarily from transient increases in HIV viral loads following influenza vaccination observed in some studies [4-10], although the clinical significance of this phenomenon is unclear. Furthermore, the efficacy of influenza vaccines may be compromised by reduced antibody responses observed in some HIV infected individuals [4,11-16]. Despite these issues, the US Center for Disease Control and Prevention (CDC) recommends influenza vaccines in HIV-infected individuals [17]. Though this recommendation may be justified by an increased susceptibility to influenza, prolonged viral replication and shedding as well as longer duration of influenza symptomatology and higher influenza-related mortality rates in HIV infected individuals [18], the efficacy and clinical effectiveness of influenza vaccines in HIV-infected individuals is not established. In this paper, the current evidence is reviewed to determine the efficacy and effectiveness of influenza vaccines in preventing influenza illness in HIV infected individuals.

## Methods

### Search strategy and selection criteria

We searched for all trials that assessed the efficacy or effectiveness of influenza vaccine in HIV-infected individuals of all ages. Databases searched included MEDLINE (1966 to December 2005), OVID (1966 to December 2005), EMBASE (1974 to December 2005), WHO website, Cochrane Acute Respiratory Infections (ARI) Group Trials Register, Cochrane Central Register of Controlled Trials (CENTRAL) and the clinical trials register (clinicaltrials.gov). We used the following key search terms: influenza vaccine, flu vaccine, HIV, influenza cases, efficacy and effectiveness. We included studies in the analysis if they assessed the efficacy or effectiveness of any influenza vaccine given in any dose, preparation, or time schedule; compared with placebo or with no intervention and in any geographical location in HIV infected individuals. Though eligible for inclusion, we did not identify any study published in any language other than English. We evaluated the efficacy of the vaccine, using as outcome the occurrence of culture- or serologically-confirmed influenza, and effectiveness using as outcome, clinical influenza-like illness during the influenza season following vaccine administration. Randomized clinical trials, cohort and case-control studies were eligible for inclusion in this analysis. Studies had to report enough data to estimate vaccine efficacy for prevention of clinically and/or laboratory confirmed cases of influenza.

Two authors independently applied the inclusion criteria to identify all relevant articles which were also reviewed for further references. We extracted the following information from the identified studies: year of study, location, study design, type of vaccine, patients' characteristics, and outcomes. When more than one case definition of influenza was used, we extracted data for all outcome definitions. If relevant information regarding the study design, patient characteristics or outcomes was unavailable, or if doubt existed about duplicate publications, we contacted authors to obtain the necessary information. We resolved discrepancies by consensus.

### Statistical analysis

All statistical calculations were performed using STATA, Version 8.0 (StataCorp, College Station, Texas, USA). We computed for each study, the risk difference (RD), number needed to vaccinate (NNV) and vaccine efficacy/effectiveness. Pooled estimates of the RD were obtained using fixed and random effects model by the DerSimonian and Laird method [19]. The heterogeneity chi-square statistic was used to assess the degree of heterogeneity between studies. Because heterogeneity tests have low power especially with a small number of studies, we used an alpha-level of 0.20 to reject the null hypothesis of homogeneity. To allow for heterogeneity, the summary estimate was obtained using a random effect model. We could not explore the sources of heterogeneity by conducting subgroup analyzes or meta-regression because of the small number of studies identified. We assessed the presence of publication bias by examining Funnel plots for asymmetry, and using the Begg's rank correlation test and Egger regression asymmetry test [20-23].

## Results

We identified 6 studies that reported the risk of influenza in HIV-infected individuals. However, for this meta- analysis we only included 4 studies, because one study was a duplicate [24] and the other study did not have enough information to calculate an effectiveness measure [25]. In the latter study, 72 HIV-infected individuals were vaccinated with a single dose of 15 μg of the 1998–1999 season influenza vaccine (A/Sydney/5/97 (H3N2), A/Beijing/262/95 (H1N1), B/Beijing/184/93). There were 18 cases (25%) of influenza-like illness characterized by abrupt onset of fever (> 39°C), myalgia and sore throat. Since all of the individuals who were HIV positive were vaccinated, it was impossible to obtain an effectiveness measure and therefore we did not include the study in the meta-analysis.

Table 1 shows selected characteristics of the studies included in our analysis. These studies involved a total of 646 study participants. The studies were conducted in the USA (n = 2), Japan (n = 1) and Italy (n = 1), between 1995 and 2002. The age of the participants ranged from 20 to 78 years. We did not identify studies that included HIV-infected children as participants. The majority of the study participants were male comprising 82% (n = 532) of all the subjects included in this meta-analysis. Except for one study, which reported a median CD4 cell count of 149 cells/μl [4], the majority of individuals in the other studies were not severely immunocompromised with the median CD4 cell count being above 400 cells/μl. The proportion of individuals receiving highly active antiretroviral therapy (HAART) across the studies ranged from 56% to 96%. All of the studies were conducted in a health facility setting and the follow-up period ranged from 3 months to 2 years. Only one study [26] randomly assigned study participants to vaccine or placebo groups and masked the subjects from the treatment allocation. The rest of the studies did not randomly assign individuals and did not provide a placebo for the control arm. Three studies used a dose of 15 μg of vaccine but one study did not report the dosage of vaccine used [4]. The vaccines in all the studies included more than one viral strain.

**Table 1:** Characteristics of studies evaluating the effectiveness of influenza vaccine in HIV infected Individuals

**Study, location and reference**	**Number of Patients**	**Study Design**	Age * (years)	CD4 Cell Count**	**Vaccine strains**		**Comparator**
Tasker 1999, USA, [26]	102	Randomized double blind, placebo controlled	33	403.1	A/Johannesburg/33/94 A/Texas/36/91 B/Harbin/07/94)	(H3N2), (HlNl),	Saline
Fine 2001, USA, [4]	71	Outbreak investigation	38	149	A/Nanchang/933/95(H3N2), A/Texas/36/91(H1N1), B/Harbin/07/94, York/83/97(H3N2)	A/New	None
Raineri 2005, Italy, [34]	145	Prospective nonrandomized	20–69	--	INFLEXAL V, Berna		None
Yamanaka 2005, Japan, [27]	328	Prospective nonrandomized	40.8	379	A/New Caledonia/20/99 A/Panama/2007/99 B/Shanton/7/87	(H1N1), (H3N2),	None

*- mean or range; **-median values (mean used when median was not reported[26])

**Figure 1:**
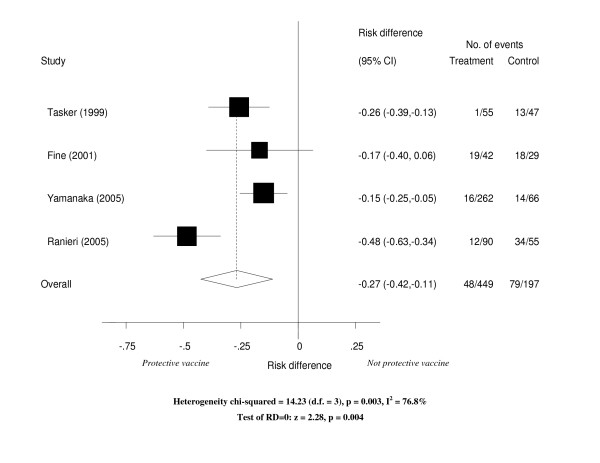
Forest plot of studies of the effectiveness of influenza vaccines in HIV-infected individuals.

Table 2 shows the total number of subjects in the vaccine and in the control groups and the number of events in each group for each study. Cases of influenza occurred less frequently in the vaccinated group compared to the non-vaccinated group. All but one study reported a statistically significant protection against influenza by vaccine. The RD ranged from -0.15 (95% confidence interval -0.25 to -0.05) to -0.48 (95% confidence interval -0.63 to -0.34) (Figure 1). In these studies, between 3 and 7 HIV-infected subjects needed to be vaccinated to prevent one case of influenza. However, vaccine effectiveness ranged from 27% to 78% (Table 2). It is worth noting that unlike the RD, VE is a measure of the difference in risks between vaccinated and unvaccinated, accounting for the observed (baseline) risk of disease in people who did not receive vaccine. Thus, despite having the lowest RD, the study conducted in Japan [27] had a relatively high VE of 70%.

**Table 2:** Effectiveness of influenza vaccines in HIV-infected patients

**Study**	**Outcome Studied**	**Vaccine**	**Control**	**Risk Difference (95% C.I.)**	NNV*	Vaccine effectiveness **
Tasker 1999 [26]	Symptoms of respiratory illness, at least 4 fold increase in antibody titre, viral culture	16/55	23/47	-0.198 (-0.387, -0.01)	5	41%
Fine 2001, [4]	Influenza like illness or a 4 fold increase in antibody titers or isolation of influenza virus	19/42	18/29	-0.168 (-0.404, -0.067)	6	27%
Raineri 2005, [34]	Influenza illness	12/90	34/55	-0.485 (-0.632, -0.337)	3	78%
Yamanaka 2005, [27]	Influenza like symptoms with at least 4 fold increase in antibody titre or viral isolation in culture	16/262	14/66	-0.151 (-0.255, -0.048)	7	71%

C.I.: Confidence Interval; NNV: * Number needed to vaccinate (= 1/RD); ** Vaccine effectiveness = 100*(1-risk ratio)

Ranieri et al [34] only evaluated the effectiveness of vaccines in preventing clinical cases of flu and reported the highest risk reduction (RD of -0.48). The other three studies [4,24,26] evaluated both the efficacy of vaccination for prevention of laboratory confirmed (serologically and/or by culture) cases and the effectiveness of vaccines in preventing clinically confirmed cases of influenza (Table 2). Only Tasker et al. reported the number of outcomes separately according to the different case definitions of influenza used in the study [26]. Vaccine significantly reduced the occurrence of influenza for all the three case definitions. The RD was -0.11 (95% confidence interval -0.20 to -0.02) for cases diagnosed using viral culture; -0.20 (95% confidence interval -0.32 to -0.07) for cases diagnosed using a 4-fold increase in antibody titers and -0.26 (95% confidence interval -0.39 to -0.12) for cases diagnosed using either culture or serology. The summary effect measure of effectiveness of the vaccines included clinical and laboratory diagnosis of flu cases. Overall, vaccinating HIV-infected individuals resulted in a significant reduction in the occurrence of influenza cases with a RD of -0.25 (95% confidence interval ranging from -0.43 to -0.08) (Figure 1). This overall estimate should be treated with caution as there was evidence of heterogeneity across studies (chi-square = 14.23, p-value = 0.003).

We assessed publication bias graphically (using a funnel plot) and statistically. The funnel plot displays a plot of the risk difference versus the reciprocal of the standard error. However, because of the small number of studies included in this meta-analysis, it was difficult to visually determine the absence or presence of asymmetry by examining this plot. Nevertheless, both the Begg's rank correlation (p-value = 1.00) and Egger's regression tests (p-value = 0.819) did not show any evidence of publication bias.

## Discussion

This study represents the first published systematic review with meta-analysis of the effectiveness of influenza vaccines in HIV-infected persons. Although we identified a limited number of studies, this review suggests a reduction in the incidence of influenza following vaccination. The magnitude of the reduction is however moderate as evidence by the vaccine effectiveness computed for each of the studies. The vaccine effectiveness was similar to that reported in HIV-negative individuals and in the elderly [28,29]. Even though influenza vaccine is generally recommended for the latter, in HIV-infected persons, the safety of vaccines in each individual needs to be considered before its administration. In HIV-infected persons, influenza vaccines may result in an increase in HIV plasma level and/or a reduction in CD4 cell counts. These changes, in most cases, have been transient [30], but warrant serious consideration. The evidence of effect heterogeneity in our analysis further supports the need for individual considerations in deciding to administer influenza to each HIV-infected person. Differences in study design, vaccine composition, or study population could be the reason for heterogeneity. However these could not be explored in this analysis because of the limited number of studies identified.

Most previous studies of influenza vaccines in HIV-infected persons focused on their immunogenicity rather than their clinical effectiveness [11-14,24,31-33]. These immunogenicity studies confirmed the production of protective antibodies in HIV infected persons even though the antibody levels in HIV infected persons appear to be lower to those in HIV-negative persons. Although the effectiveness of vaccines is mediated by the immune response, the presence of an immune response may not always guarantee clinical effectiveness in preventing infection and/or reducing disease severity following infection. The results of clinical studies reviewed here suggest that immune response levels in some HIV-infected persons may be sufficiently protective against clinical disease. Furthermore, the highest risk reduction reported in Ranieri et al.'s study indicates that vaccine may be more effective in preventing clinical disease than it is in preventing infection per se.

We only identified one randomized study. The three other studies were liable to selection bias and/or confounding. Due to the few number of subjects included in each study, we were not able to assess the effect of the stage of HIV disease (HIV viral load and or CD4 levels) or antiretroviral treatment on the effectiveness of vaccines. In addition, because these studies were conducted in developed countries with predominantly male study populations, their results may not be generalizable to the majority of HIV-infected men and women who live in developing countries. With the threat of a global influenza pandemic, there is an urgent need to evaluate the effectiveness of influenza vaccines in a larger number of representative HIV-infected persons throughout the world.

## Conclusion

Current evidence suggests that influenza vaccines are effective, albeit moderately, in reducing the incidence of influenza in HIV-infected individuals. Vaccination is thus a potentially useful intervention and should be considered in the care of HIV-infected individuals. Further studies of clinical effectiveness recruiting larger numbers of HIV-infected subjects and in populations most affected by HIV are, however, required.

## Abbreviations

AIDS: Acquired Immune-Deficiency Syndrome

CI: Confidence interval

HAART: Highly Active Anti-Retoviral Therapy

HIV: Human Immune-deficiency Virus

NNV: Number needed to vaccinate

RD: Risk difference

USA: United States of America

VE: Vaccine effectiveness

WHO: World Health Organization

## Competing interests

The author(s) declare that they have no competing interests.

## Authors' contributions

JA and LK conducted the literature search and data analysis. All authors wrote and reviewed the manuscript.

## Pre-publication history

The pre-publication history for this paper can be accessed here:


